# PVformer: Pedestrian and Vehicle Detection Algorithm Based on Swin Transformer in Rainy Scenes

**DOI:** 10.3390/s22155667

**Published:** 2022-07-28

**Authors:** Zaiming Sun, Chang’an Liu, Hongquan Qu, Guangda Xie

**Affiliations:** 1 School of Control and Computer Engineering, North China Electric Power University, Beijing 102206, China; 2 Information College, North China University of Technology, Beijing 100144, China; liuchangan@ncut.edu.cn (C.L.); qhqphd@ncut.edu.cn (H.Q.); 3 School of Electrical and Control Engineering, North China University of Technology, Beijing 100144, China; 2020413010103@mail.ncut.edu.cn

**Keywords:** Swin Transformer, image deraining, pedestrian and vehicle detection

## Abstract

Pedestrian and vehicle detection plays a key role in the safe driving of autonomous vehicles. Although transformer-based object detection algorithms have made great progress, the accuracy of detection in rainy scenarios is still challenging. Based on the Swin Transformer, this paper proposes an end-to-end pedestrian and vehicle detection algorithm (PVformer) with deraining module, which improves the image quality and detection accuracy in rainy scenes. Based on Transformer blocks, a four-branch feature mapping model was introduced to achieve deraining from a single image, thereby mitigating the influence of rain streak occlusion on the detector performance. According to the trouble of small object detection only by visual transformer, we designed a local enhancement perception block based on CNN and Transformer. In addition, the deraining module and the detection module were combined to train the PVformer model through transfer learning. The experimental results show that the algorithm performed well on rainy days and significantly improved the accuracy of pedestrian and vehicle detection.

## 1. Introduction

Image object detection in bad weather is a key issue for autonomous driving systems. Image quality deteriorates under rainy conditions, resulting in low contrast, low visibility, blurred edges, and other issues. Furthermore, the object detection accuracy cannot be guaranteed. With the development of science and technology, intelligent transportation has attracted great attention from all countries. Having a high-precision visual detection system is the cornerstone of the development of autonomous driving. A robust computer vision system needs to adapt various conditions including bad weather. However, most of the vision algorithms can only work on clean images. Therefore, in-depth research on how to enhance the quality of degraded images has important theoretical and practical application value for improving the performance of object detection in corresponding scenes.

Although research on single image deraining or pedestrian and vehicle detection has been extensive [1,2], there have been few studies on detection algorithms with the rainy condition. For the part of single image deraining, the techniques proposed at home and abroad can be roughly divided into the following three categories: the deraining methods based on image filtering; domain prior [3,4,5]; and deep learning [6,7,8]. Among them, the methods based on image filtering and domain priors generally use artificially designed features. The purpose of deraining is achieved through a model-driven approach. For example, to simultaneously accommodate different types of noise, Chang et al. (2017) [9] provided total variation in the compositional direction and low-rank prior for the image layer, after detailed analysis of noisy and clean images in both the local gradient and non-local domain. Li et al. [10] proposed a method of applying priors to both the background and rain layers. These priors are based on Gaussian mixture models and can accommodate multiple orientations or scales of the rain streaks. The robustness and accuracy of these algorithms are mostly difficult to meet the requirements of practical applications. Moreover, the deep learning-based deraining methods combine the deep neural network and big data with strong modeling capabilities in recent years, and establishes an end-to-end mapping between the original image and the corresponding background image through a data-driven model, thereby improving the visual effect of degraded images on rainy days.

For detection tasks, with the development of deep learning, a large number of excellent object detection algorithms have emerged. Since 2012, the convolutional neural network (CNN) [11] has been the dominant player in vision tasks [12,13]. At present, image object detection algorithms based on deep learning are mainly divided into two categories. One is the regression methods, represented by YOLO [14], SSD [15], RetinaNet [16], etc. The other is the two-stage method based on the candidate box represented by R-CNN [17], Fast R-CNN [18], and Faster R-CNN [19]. Zhang et al. [20] utilized ResNet [21] as the backbone of the detection network to jointly predict the location and category (i.e., pedestrian or not) of bounding-box proposals. This method can obtain better pedestrian detection accuracy by improving the feature extraction ability of the network. Ghosh et al. [22] proposed an end-to-end road vehicle detection method based on an improved Faster RCNN, where ROIs are generated using several varying sized RPNs, and therefore it is able to detect varying sized vehicles. CNNs are very beneficial in extracting local information, but they lack the ability to extract long-range features from global information.

In the field of power system, Transformer is regarded as one of the crucial parts of the electrical power transmission and distribution system [23,24,25]. In the field of computing, Transformer was born in the field of natural language processing (NLP) [26,27,28]. With the fusion of computer vision and NLP, various efficient structures have emerged. The application of Transformer to computer vision tasks has also recently become one of the most popular areas of research. Unlike CNN, Transformer is powerful in focusing on global information modeling [29]. Parmar et al. [30] first applied Transformer to image generation in 2018 and proposed Image Transformer. Chen et al. [31] converted images into a one-dimensional sequence as input and trained a sequence Transformer to achieve competitive results with neural networks on image classification tasks by performing auto-regressive predictions on pixels. Dosovitskiy et al. [29] proposed a simple Transformer that does not rely on CNN, called Vision Transformer (ViT). ViT can be directly applied to sequences of image patches and achieve state-of-the-art performance on multiple image recognition benchmark datasets. By 2020, Carion et al. [32] combined CNN with Transformer to propose a complete end-to-end object detection framework DETR. Transformer was applied to object detection for the first time when Tan et al. [33] introduced the Swin Transformer into the field of image deraining for the first time. By improving the basic block of the Swin Transformer, its performance and potential in the field of image deraining can be studied.

For pedestrian and vehicle detection, the special scene of rainy days makes the detection more difficult. For example, in rainy images, small objects in the distance occupy fewer pixels and are easier to be ignored in feature extraction process. In the case of heavy rain, the combination of the rain pattern and background will lead to difficulties in the feature extraction of the detection model. There will be false and missed detection. In this paper, a new rainy image pedestrian and vehicle detection network is presented. The image quality in the rainy condition was enhanced and the object detection performance in the corresponding scenes was improved. Our contribution is divided into four parts:

(1) This paper proposes a pedestrian and vehicle detection algorithm for rainy days that combines deraining and detection. The model can be obtained by transfer learning, which greatly improves the object detection accuracy of rainy images.
(2) A deraining model (DEST) was designed based on the Swin Transformer block. In addition, a four-branch feature fusion network structure was proposed to improve the quality of feature extraction.
(3) According to the characteristics of rainy images, a local perception Swin Transformer (LPST) backbone is proposed by combining the CNN and Transformer, which enhances the local perception ability of the algorithm for rainy images.
(4) At present, in light of the limited resources of rainy images, this paper creates and annotated a new dataset named Rain-PV, which includes 12,844 images selected from the existing object detection and public rain datasets.

## 2. Related Work

### 2.1. Single Image Deraining

For the image deraining task, widely used traditional methods include dictionary learning [34], low rank model [35], and the Gauss Mixture Model (GMM) [36]. Kang et al. [37] proposed a single-image-based rain removal framework. Combined with bilateral filtering, the traditional morphological method was used to decompose the high and low frequency parts of the image. Then, the rain pattern noise in the high frequency component was processed. Finally, the background information in the low frequency components was fused to obtain a clean image. Luo et al. [38] proposed a single image rain removal algorithm based on dictionary learning, which mainly used dictionary learning and sparse coding to separate the rain layer. The traditional image deraining algorithms usually detect and remove the rain patterns through predefined models, so it is difficult to achieve a satisfactory effect in detail restoration.

For the deraining method of deep learning, Zhang H et al. [39] proposed an image deraining method based on the conditional generative adversarial network (CGAN), in which the generator used a densely connected symmetric network. In order to effectively use the features from different levels, the discriminator adopts a multi-scale output. Then, the local and global information of the image is fully captured to judge its authenticity. Yasarla R et al. [40] proposed a deraining network based on image quality. The network guides the optimization of network weights based on the quality estimates at each location and the confidence of the remaining rain pattern information (residual maps). Yasarla R et al. [41] proposed a semi-supervised transfer learning network to achieve image deraining. The Kullback–Leibler divergence is also used for constraint training to make the distribution of synthetic rain closer to that of real rain. Non-parametric methods are used to generate the supervision of unlabeled image data so that unlabeled real images can be integrated in the training.

### 2.2. Pedestrian and Vehicle Detection Methods

For pedestrian and vehicle detection, Hu et al. [42] proposed a cascade vehicle detection method combining multi-feature fusion and convolutional neural network (CNN), which had good robustness in a complex driving environment. Cai et al. [43] proposed a multi-scale CNN (MS-CNN) network that could detect vehicles of different scales by using the information from different feature map resolutions. For multi-scale vehicle detection, Zhao et al. [44] proposed an FPES method to enhance the quality of feature pyramids. Chen et al. [45] used the visual attention mechanism to extract the object candidate regions and generated the detection sub-window in them. Then, the vehicle detection was realized by a cascade classifier. Shao et al. [46] effectively solved the occlusion problem of human detection in a highly crowded environment. Wanchaitanawong et al. [47] proposed a multi-modal Faster-RCNN. This algorithm was robust to large imbalances and enhanced the ability to detect multi-scale pedestrians. Xiao et al. [48] proposed an improved method for pedestrian detection using the visual attention mechanism and semantic computation. The total significance map was obtained by building a static visual attention model to complete the pedestrian detection. To improve the imbalance between accuracy and speed, Ma et al. [49] proposed a pedestrian detection method based on the multi-scale attention mechanism of a convolutional neural network.

### 2.3. Vision Transformer

The transformer structure can be represented as an encoder and decoder. The encoder and decoder mainly consist of self-attention and feed-forward networks, while self-attention consists of scaled dot-product attention and multi-head attention. The mathematical expression of attention is as follows:

(1) $$Attention(Q,K,V)=Softmax\left(QK^{T}/\sqrt{d_{k}}+B\right)V$$

where $Q,K,V\in {\mathbb{R}}^{M^{2}\times d}$ represent the query, key and value matrix, respectively. $M^{2}$ represents the number of patches in the window. *d* represents the dimension of the query or key. *B* is taken from the bias matrix $\hat{B}\in {\mathbb{R}}^{\left(2M-1)\times (2M-1\right)}$.

Specifically, the Swin Transformer, proposed by Liu et al. [50], is an innovative vision Transformer model. Its hierarchical structure has the flexibility to model at various scales and has powerful image processing capabilities. The Swin Transformer adopts the shifted window to make the network computation increase linearly, which accelerates the reasoning speed of the network. Tan et al. [51] proposed a dual branch deraining network based on Transformer. This model was able to capture channel attention more finely and focus on regions of high rain streaks density to achieve a better deraining effect. Valanarasu et al. [52] proposed a novel Transformer encoder architecture that used Transformer blocks within a patch to enhance attention inside the patch, effectively removing smaller weather degradations. Xu et al. [53] solved the problem of unsatisfactory edge detail segmentation by improving the Swin Transformer and improved the detection accuracy of the network for small scale objects.

## 3. The PVformer Model

### 3.1. Overall Architecture

**Figure 1:**
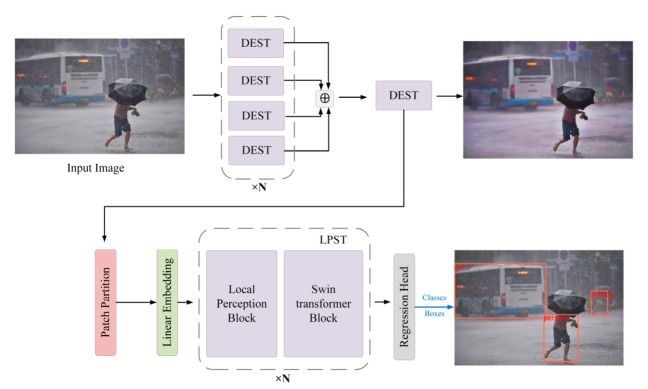
The overall architecture of pedestrian and vehicle detection on rainy days. The new network could accurately complete the task of rainy image object detection and image deraining.

In this section, we introduce a rainy day pedestrian and vehicle detection network based on the Swin Transformer, called PVformer. Its network structure is shown in Figure 1. We constructed an end-to-end learning network to perform the object detection task of a rainy image. The Swin Transformer was selected as the design prototype of the backbone network. Considering the complexity and efficiency, the Swin-B version was adopted. The whole network can be divided into two parts: image deraining and object detection. The image deraining part is composed of an efficient four-branch fusion module, which extracts features through a feedforward network to achieve the purpose of deraining. In order to use more context without losing local details, the local perception Swin Transformer (LPST) module is designed in the pedestrian and vehicle detection part. This module enhances the local perception of the network and improves the detection accuracy of small objects.

### 3.2. Deraining Module

**Figure 2:**
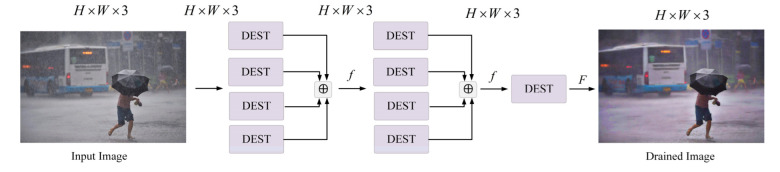
The derain model.

**Figure 3:**
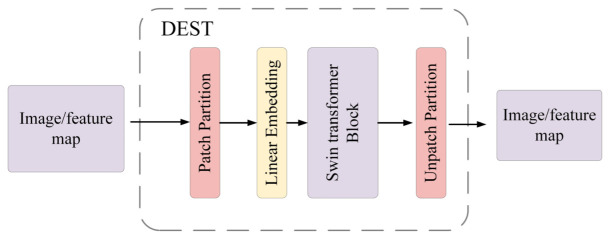
The derain Swin Transformer (DEST) block.

For the image deraining, we adopted a four-branch fusion network of the Swin Transformer. It utilizes the powerful feature representation capability of the Swin Transformer to construct a multi-scale feature vector model, as shown in Figure 2. The model feeds the image into a four-branch fusion block. Each branch consists of the derain Swin Transformer (DEST) modules. The DEST structure is shown in Figure 3.

For the four-branch fusion network composed of DEST, the mathematical expression is as follows:

(2) $$F=f(\sum_{i=1}^{n}f_{i}(x))$$

where $f_{i}(\cdot )$ denotes the operation of the DEST module; x denotes the input of the module; *n* = 4 denotes four DEST operations. Through learning $f_{1}$ to $f_{4}$, it can adaptively learn different features. This structure is similar to the multi-head attention mechanism in self-attention, except that we sum up the extracted features rather than contact them. After two cycles, the added features are fused by *F* to further realize the extraction of deep and shallow features in the network.

Inspired by the Swin Transformer, our DEST module design is shown in Figure 3. According to the structure of the Swin Transformer, we added an Unpatch Partition operation after the Swin Transformer block. This is the opposite operation of patch partition, so the output image or feature is the same size as the input image.

### 3.3. Pedestrian and Vehicle Detection Network

**Figure 4:**
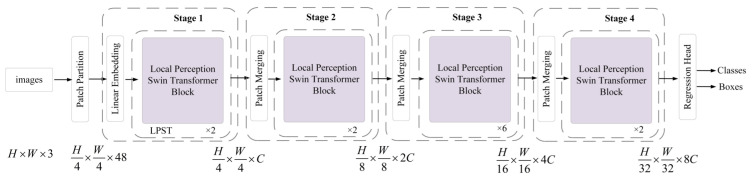
The architecture of the pedestrian and vehicle detection network.

This section focuses on the network structure of the pedestrian and vehicle detection section. Compared with the most commonly used CNN-based detection algorithm, this paper used a hierarchical vision The Transformer object detection algorithm was based on the shifted window, which is the latest Swin Transformer [50] framework. The model feeds the features extracted from the deraining part to the LPST backbone network. The detection algorithm flow is shown in Figure 4. Similar to the Swin Transformer, there are two, two, six, and two blocks per stage.

First, given a derained image I of size H×W×3, the input image H×W×3 is divided into a set of non-overlapping patches by patch partition. The size of each patch is 4×4, the feature dimension is 4×4×3, and the number is H/4×W/4. Then, by linear embedding, each patch is treated as a “token” and its feature is set as a concatenation of the raw pixel RGB values. After the feature dimension of the divided patch token is changed to 4×4×C, it is fed into LPST blocks at different stages for encoding. Then, the input is merged according to the adjacent patches of 2×2, so the number of patch blocks becomes H/8×W/8 and the feature dimension becomes 4C. This step is repeated N times in each stage until the number of patch blocks becomes H/32×W/32 and the feature dimension becomes 8C. Then, the regression head is sent for object classification and positioning regression. Each stage consists of a patch merging block or a linear embedding block and some local perception Swin Transformer blocks.

#### Local Perception Swin Transformer (LPST)

**Figure 5:**
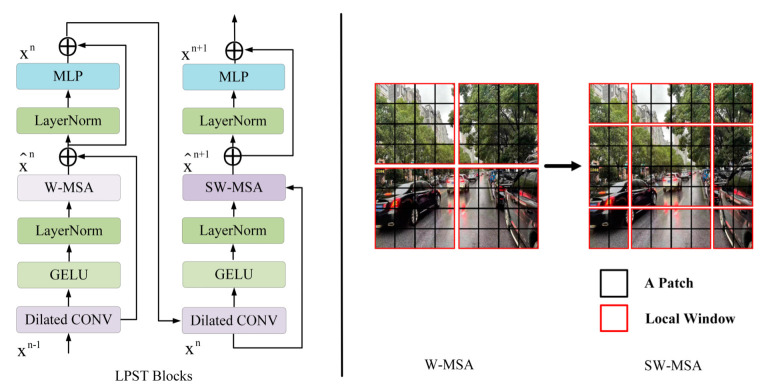
An illustration of the local perception Swin Transformer (LPST) block. W-MSA and SW-MSA are multi-head self-attention modules with regular and shifted windowing configurations, respectively.

Positional encoding in a transformer can easily fail to detect local correlations and structural information in images. The Swin Transformer uses a window-based hierarchy to solve the scale and computational complexity problems of high-resolution images. One Swin Transformer block consists of a shifted window based MSA module, followed by a 2-layer MLP with GELU nonlinearity in between. Each multi-head self-attention module and each MLP module are preceded by a layer normalization (LN) operation, followed by a residual connection. Unlike the conventional MSA module, the Swin Transformer block is constructed based on shifted windows. When two successive Transformer blocks are connected in series, self-attention layers are, respectively, multi-head self-attention modules with regular and shifted windowing configurations, that is, W-MSA and SW-MSA. The window partition scheme is shown on the right in Figure 5, where the red area represents the window and the black area represents the divided patch block. The conventional division is to divide the feature map with the size 8 × 8 into 2 × 2 windows, each window has 4 × 4 patches, and then calculate the self-attention within each window. The partitions divided by the shifted windows are moved, as shown on the right in Figure 5, new windows are generated, and they cross the boundaries of regular windows. The purpose is to provide the connection of information between adjacent windows. By introducing local ideas, only local relationships are modeled for each layer. At the same time, the width and height of the feature map are continuously reduced, thereby expanding the receptive field and maintaining the efficient calculation of non-overlapping windows.

The Swin Transformer builds a hierarchical Transformer and implements attention operations on each window without overlapping. However, the Swin Transformer is limited in its ability to encode contextual information. To enhance the network’s learning about local correlation and structure information, we propose a local perception Swin Transformer (LPST). As shown to the left of Figure 5, each LPST block is composed of two consecutive improved Swin Transformer blocks. A layer of 3 × 3 dilated convolution (dilation = 2) and a GELU activation function is added in front of the LayerNorm layer.

Dilated convolutions can enlarge the receptive field of spatial images, thus encoding the global multi-scale context information better, which is very useful for detection and recognition. In addition to the size of the convolution kernel, compared with ordinary convolution, the dilated convolution also has a dilation rate parameter. This parameter is mainly used to indicate the size of the dilation. The similarity between these two convolutions is that the size of the convolution kernel is the same, so the number of parameters in the neural network remains unchanged. The difference is that the dilated convolution has a larger receptive field and improves the expressive power of the network model. The receptive field is the size of the convolution kernel as seen on the image, for example, a 3 × 3 convolution kernel has a receptive field size of 9. If it is a dilated convolution (dilation = 2) with the same kernel size, the receptive field is 7 × 7. Dilated convolutions can effectively increase the receptive field of the network model when the parameters and amount of calculation are equal.

### 3.4. Loss Function

For image deraining, our loss function consists of two parts:

(3) $$Loss_{L_{2}}=L_{2}(PVformer(O),B)$$

(4) $${\;Loss}_{SSIM}=SSIM(PVformer(O),B)$$

(5) $${Loss}_{all}=\alpha \times {Loss}_{L_{2}}+\beta \times \left(1-{Loss}_{SSIM}\right)$$

For Equation (3), *O* is the rainy image and *B* is the corresponding label. The $L_{2}$ loss function is also known as the MSE (mean square error), which is the square of the difference between the predicted value and the true value. For Equation (4), the structure similarity index measure (SSIM) is a metric used to evaluate the structural similarity of the content of two images. Select the negative SSIM as the loss function. Equation (5) represents our overall loss to the rain. Set α and *β* to 0.2 and 4, respectively, representing the coefficients of $L_{2}$ loss and the SSIM loss.

For the detection and training stage of the pedestrian and vehicle, the cascade mask R-CNN regression module was used. The module includes a classifier $h_{x}$ and a regressor $f_{x}$. The classifier allocates an image block x to one of the M+1 classes. The extra class represents the background class, and the loss of the classifier is set as:

(6) $$R_{cls}[h]=\sum_{i=1}^{N}L_{cls}\left(h\left(x_{i}\right),y_{i}\right)$$

where h(x) is the M+1 dimensional estimate of the class posterior distribution; $x_{i}$ is the network input; $y_{i}$ is the class number; $L_{cls}$ is the cross-entropy loss; and N is the batch size. In label prediction, because the bounding box usually includes an object and some background, it is difficult to determine the correctness of the detection, so it is solved by the IoU index. If it is higher than the threshold u, the image patch x is responsible for the prediction of the object. It is assumed that the category label of x is a function of u, and inferred from u:

(7) $$y=\{\begin{array}{cc} g_{y}, & IoU(x,g)\geq u\\ 0, & \;otherwise\; \end{array}$$

where $g_{y}$ is the ground truth box position label; g is the real category. The task of the regressor is to use the regressor f(x,b) to return a candidate box b to the position of the real object box g. A box contains the four coordinates of $\left(b_{x},\;b_{y},\;b_{w},\;b_{h}\right)$, and the loss of the regressor is set as:

(8) $$R_{loc}[f]=\sum_{i=1}^{N}L_{loc}\left(f\left(x_{i},b_{i}\right),g_{i}\right)$$

where $L_{loc}$ is the $L_{2}$ loss; in addition, the IoU threshold is optimized at each training stage t, and the optimized cascade loss is defined as:

(9) $$L\left(x^{t},g\right)=L_{cls}\left(h_{t}\left(x^{t}\right),y^{t}\right)+\lambda \left[y^{t}\geq 1\right]L_{loc}\left(f_{t}\left(x^{t},b^{t}\right),g\right)$$

where $b^{t}=f_{t-1}\left(x^{t-1},b^{t-1}\right)$; g is the ground truth box of $x^{t}$; λ=1 is the trade-off coefficient; [·] is the index function; $y^{t}$ is the label. Cascade loss guarantees that effectively trained detectors continuously improve the detection of locations. In inference, by applying the same cascade process, the quality of the proposal bounding box will be sequentially improved so that high quality object detection is achieved.

## 4. Experiments and Analysis

In this section, we evaluate the validity of the PVformer model on synthetic and rainy datasets through the experiments. In what follows, we explain the datasets, experiment environment, implementation details, experimental settings, results, and comparison with state-of-the-art methods.

### 4.1. Dataset

For the image deraining task, we used Rain100L, Rain100H [54], Rain800 [39], Raindrop, and Attentive GAN-Data [55] for training. There were 6587 pairs of rain images, each of which contains a rainy image and a real background image. For the detection dataset of pedestrians and vehicles on rainy days, we obtained images from the deraining task datasets: the public dataset RID&RIS [56], KITTI [57], UA-DETRAC [58], COCO [59], PASCAL VOC, and the Internet. There was a total of 12,844 rainy images including real and synthetic rain. According to the Cityscapes [60] annotation protocol, the category is marked as a person and car. For the bus and truck classes in the vehicle, we uniformly marked them as cars, forming a new dataset named Rain-PV. The number of cars and person instances were 46,241 and 31,466 respectively. We used this dataset for training, which was divided into three parts: the training, validation, and test sets with a ratio of 7:1:2. To test the real scene, we collected another 150 real rainy images and labeled them for evaluation, named Real Rain-150.

### 4.2. Experiment Environment

The details of the experimental environment are shown in Table 1:

### 4.3. Evaluation Metrics for Detection

Commonly used evaluation indices of detection algorithms include precision (P), recall (R), average precision (AP), mean AP (mAP), and frames per second (FPS). True Positives (TP): The set of correctly detected objects as true positives. False Positives (FP): The set of falsely detected objects of false positives. False Negatives (FN): The set of objects which are not detected by the detector as false negatives.

The precision is now the ratio between the number of *TP* relative to all of the predicted objects:

(10) $$Precision=\frac{\left|TP\right|}{\left|TP|+|FP\right|}$$

The recall is defined as the ratio of detected objects (*TP*) relative to the number of all objects in the dataset:

(11) $$Recall=\frac{\left|TP\right|}{\left|TP|+|FN\right|}$$

Average precision (*AP*) is the area under the precision–recall curve. For the pedestrian and vehicle detection performance, *mAP* is the average of *AP* values of all categories. The mAP is the overall measurement of the average precision of detection objects. Its calculation formula is as follows:

(12) $$mAP=\frac{\sum AP(C)}{N(classes)}$$

where *classes* is the number of categories. Usually, the execution speed of the detector is evaluated by processing frames per second (FPS), namely, real-time performance. If the FPS value is larger, the real-time performance of the detector is better.

### 4.4. Implementation Details

**Figure 6:**
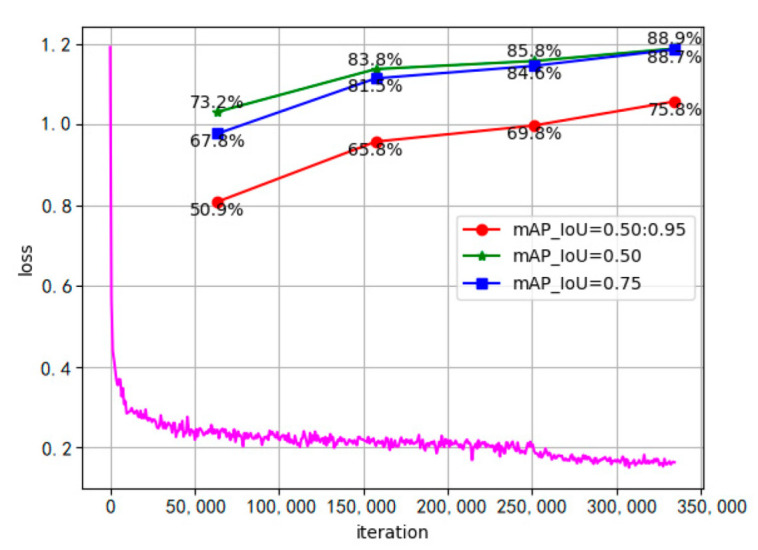
The PVformer training process and loss changes.

First, we used the classical adaptive moment estimation optimizer (Adam) to train the deraining model. The initial value of the learning rate was $1\times 10^{-3}$. When the number of training iterations reached 3/5 and 4/5 of the total number of iterations, the learning rate was reduced to $1\times 10^{-4}$ and $1\times 10^{-5}$, respectively. The image size of the input model was uniformly converted to 416 × 416, and the batch size was set to 5. The model was iterated 100,000 times on four RTX 2080Ti GPUs. It took a total of 23 h for the model to converge. The loss function curve is shown in Figure 6.

To train the detector, we adopted the method of transfer learning. The detection model was initialized with the deraining model and the Swin Transformer model pretrained on ImageNet, respectively. The optimizer used the AdamW algorithm based on weight attenuation, and the initial learning rate was set as $6\times 10^{-5}$. Using the linear learning rate decay method, the weight was set to decay 0.01 every 1500 iterations. In addition, random horizontal flipping, random scaling, and random photometric distortion were performed in the [0.5, 2.0] scale range for each image. The model performed 330,000 iterations on eight RTX 2080Ti graphics cards, with the first 250,000 iterations freezing the deraining model. It took 78 h for the model to converge. The loss function curve is shown in Figure 6, and the loss value eventually dropped to 0.18. In the case of IoU = 0.50, the mAP value reached 88.9%.

### 4.5. Experiment Results

#### 4.5.1. Result on Deraining

**Figure 7:**
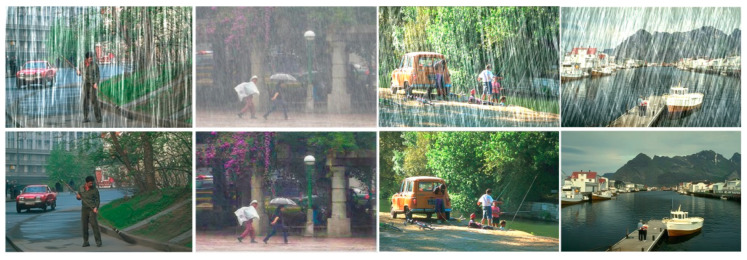
The visualization of deraining.

Deraining usually serves as preprocessing for some advanced tasks. Figure 7 shows the visualization effect of the image after deraining. The comparison showed that PVformer could both remove the rain pattern well and recover the texture details satisfactorily, this lays the foundation for the subsequent detection or performance improvement.

#### 4.5.2. Comparison of Detection Results

In order to verify the effectiveness of the algorithm proposed in this paper, we compared the experimental results with the most advanced detection models Mobilenet [61], SSD, Yolov5, Faster-RCNN, and Swin-Transformer. From the comparison of the qualitative and quantitative results, the detection performance of PVformer in this paper was better than that of other algorithms on rainy days. In addition, by testing the images on rainy days to varying degrees, the results showed that our algorithm had better robustness to varying degrees of noise.

**Figure 8:**
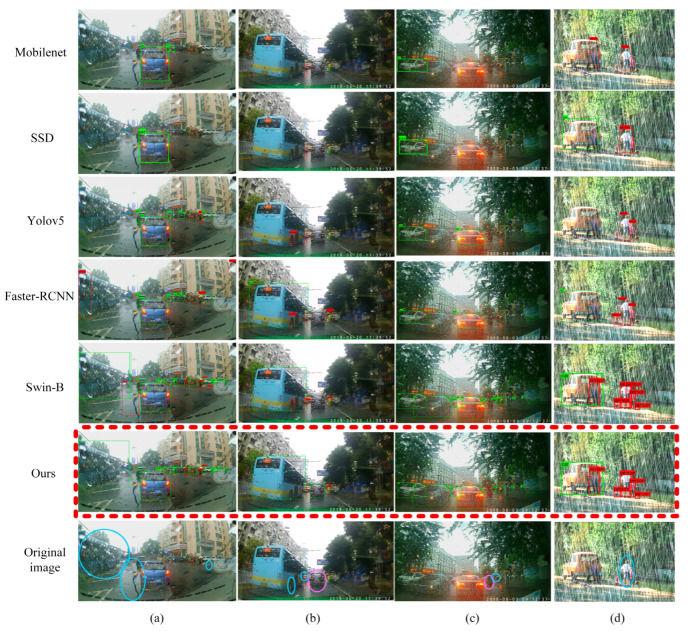
The qualitative comparisons of the detection results between the PVformer and the current best detection models on the Real Rain-150 and Rain-PV test datasets. (**a**–**c**) represent real rainy images. (**d**) represents the synthetic rainy image.

The comparison of the qualitative detection results is shown in Figure 8.

For the detection effect on rainy days, due to visual constraints, the recall rate of Mobilenet and SSD was very low. Most objects could not be detected, especially small-scale pedestrians and vehicles. Yolov5 and Faster-RCNN were slightly better than the first two algorithms, but the ratio of missed and false detection was still large. The precision and recall rate of version B of Swin Transformer (Swin-B) were significantly better than the previous four methods. We also compared Swin-B with the PVformer algorithm, focusing on the part circled in the original image, which is shown in the last row of Figure 8. Blue circles represent false detections, and pink circles represent missed detections. The comparison showed that PVformer can detect pedestrians and vehicles more accurately in rainy scenes.

The quantitative test results are shown in Table 2.

For the detection accuracy, testing on the Rain-PV test set showed that the detection accuracy of the algorithm in this paper was significantly higher than that based on the CNN. It performed even better on the Real Rain-150 test set on real rainy days, with the accuracy improved by 26.2%. This was due to the Transformer’s use of attention to capture the global contextual information, thereby establishing long-range object dependence and extracting more powerful image features. In addition, by adding the image deraining module, the detection accuracy of the Swin Transformer was improved by 4.2% on the Rain-PV dataset and 7.1% on the Real Rain-150 test set, respectively.

In terms of the detection speed, the detection time of the PVformer for a single image was 70.4ms. The detection time of our algorithm was similar to that of the Swin Transformer, but not as good as the CNN-based methods. The reason is that the amount of information in the images is much larger than that of the text data, so it is necessary to design a Transformer structure that is more suitable for the image to reduce the computational cost. This is also the next research direction of this project. In the actual detection application, the algorithm in this paper can still achieve real-time by adopting the frame interval detection method, with the detection speed of 28.5 FPS. Table 2 shows the results of comparing the performance of different detection algorithms.

**Figure 9:**
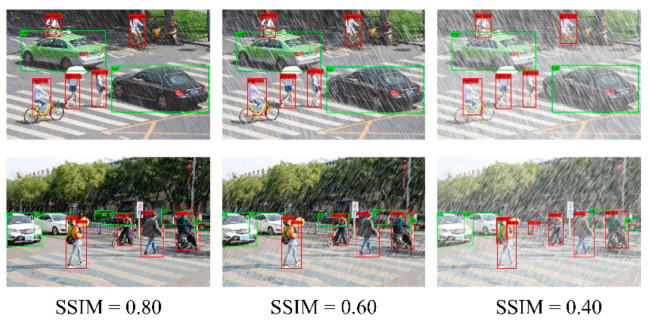
The comparison of the detection results under different SSIM values.

The SSIM value represents the degree of added noise. In order to verify the influence of noise on the proposed algorithm, artificially adding noise was used to simulate rainy days for testing. As shown in Figure 9, different SSIM values represent the strength of rain. Experiments showed that PVformer had good adaptability to different degrees of rain, reflecting strong robustness.

### 4.6. Limitations

PVformer could achieve good accuracy in pedestrian and vehicle detection tasks on rainy days. At the same time, due to the overall complexity of the model, the inference speed still needs to be improved. For example, PVformer’s single-sheet detection time was 5.1 ms longer than that of the Swin Transformer’s for the same dataset. This makes the PVformer not the first choice in scenarios where detection speed is critical. While ensuring the detection accuracy, research on a lightweight model to achieve faster inference speed will be the focus of our future research. Meanwhile, in other scenarios, our model still has a lot of room for optimization.

## 5. Conclusions

This paper proposes a pedestrian and vehicle detection network named PVformer for use in rainy scenes. The network makes full use of the powerful learning ability of Transformer to achieve end-to-end object detection in rainy scenes. Specifically, in the image deraining part, the improved Swin Transformer block was used to replace the convolution operation. A multi-branch module was designed to fuse the information from different spatial domains. In addition, according to the advantages of CNN and Transformer, a local perception Swin Transformer backbone was designed in the detection part. The purpose was to enhance the local perception ability and improve the detection accuracy of small-scale objects. Then, the deraining module was connected with the detection module, and the PVformer model was trained by the transfer learning method. The experimental results on the self-made dataset demonstrate that the mean average precision (mAP) reached 88.9%. The experiments verify the effectiveness of the entire module and framework of the PVformer network on real rainy scenes. Therefore, this study has certain theoretical and practical significance.

## Figures and Tables

**Table 1:** The experimental environment.

CPU	Intel(R) Xeon(R) Silver 4210R CPU @ 2.40GHz
GPU	NVIDIA GeForce RTX 2080Ti *4
Operating system	Ubuntu18.04
Software environment	CUDA10.1, Python3.7, OpenCV3.4, PyTorch1.6, MMDetection2.12.0, mmcv-full1.3.4

**Table 2:** The comparison results of performance of the different detection algorithms.

Test Set	Model	Backbone	mAP/%	Time/ms
Rain-PV	Mobilenet	MobileNet-224	49.4	48.8
Rain-PV	SSD	VGG-16	51.6	31.3
Rain-PV	Yolov5	Yolov5x	67.4	36.3
Rain-PV	Faster-RCNN	ResNet-101	73.0	59.4
Rain-PV	Swin-Transformer	Swin-T	84.7	65.3
Rain-PV	Ours	Derain-PVformer	88.9	70.4
Real Rain-150	Mobilenet	MobileNet-224	37.2	48.8
Real Rain-150	SSD	VGG-16	39.6	31.3
Real Rain-150	Yolov5	Yolov5x	51.7	36.3
Real Rain-150	Faster-RCNN	ResNet-101	56.1	59.4
Real Rain-150	Swin-Transformer	Swin-T	75.2	65.3
Real Rain-150	Ours	Derain-PVformer	82.3	70.4

## Data Availability

Not applicable.
